# Novel malformations: Chiari type 1 and hydrocephalus in Zhu‐Tokita‐Takenouchi‐Kim syndrome and novel *SON* variants

**DOI:** 10.1002/ccr3.6529

**Published:** 2022-12-15

**Authors:** Piero Pavone, Federica Saia, Xena Pappalardo, Massimo Barbagallo, Adriana Prato, Renata Rizzo

**Affiliations:** ^1^ Unit of Clinical Pediatrics, AOU "Policlinico", PO "G. Rodolico" University of Catania Catania Italy; ^2^ Child and Adolescent Neurology and Psichiatry, Department of Clinical and Experimental Medicine Catania University Catania Italy; ^3^ Unit of Catania, Institute for Biomedical Research and Innovation National Council of Research Catania Italy; ^4^ Unit of Pediatrics and Pediatrics Emergency Hospital "G. Garibaldi" Catania Italy; ^5^ Department of Cognitive Sciences, Psychology, Education and Cultural Studies University of Messina Messina Italy

**Keywords:** Chiari malformation type 1, developmental delay, hydrocephalus, *SON* gene, Zhu‐Tokita‐Tachenouchi‐Kim syndrome (ZTTK)

## Abstract

Zhu‐Tokita‐Tachenouchi‐Kim syndrome (ZTTK) is a recently recognized malformation syndrome presenting with craniofacial dysmorphism, developmental delay/intellectual disability, seizures, anomalies involving brain white matter, and other body‐organs. In humans, the disorder is linked to the loss‐of‐function variants in the *SON* gene (MIM# 617140). Herewith, a new case of this syndrome is reported in a 2‐year‐old Caucasian child who presented the classical clinical features of the ZTTK syndrome in association with hydrocephalus and Chiari malformations type 1 an anomaly previously unreported. Exome analysis showed a de novo heterozygous variant in SON gene. Literature review of similar cases is reported.

## Graphical abstract

Zhu‐Tokita‐Tachenouchi‐Kim syndrome (ZTTK) is a severe malformation syndrome characterized by craniofacial dysmorphism, developmental delay/intellectual disability, seizures, anomalies involving brain white matter, and other body‐organs. The disorder is linked to the loss‐of‐function variants in the *SON* gene (MIM# 617140).

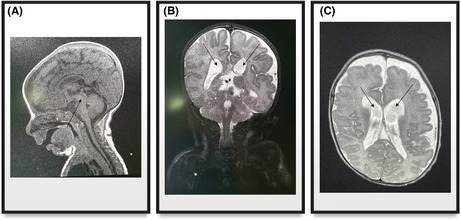

## 1 INTRODUCTION

Zhu‐Tokita‐Tachenouchi‐Kim (ZTTK) syndrome is a recently recognized intellectual deficit/malformation syndrome (ID/MS) related in humans to the loss‐of‐function variants in the *SON* gene (MIM# 617140). The first report of the syndrome dated in 2015 is due to Zhu et al.^1^ and the association with mutations in *SON* gene to Kim et al.^2^ The syndrome is characterized by a complex and various association of clinical abnormalities which include craniofacial dysmorphisms, developmental delay/intellectual disability (DD/ID), epileptic seizures, brain white matter abnormalities, and other anomalies of the systemic organs.^3^ *SON* gene is located in human chromosomal region 21q22.11 and consists of 12 exons of which exon 3 is the largest involving 82% of the entire coding region. It is a large protein consisting of 2426 amino acids and repeat sequences and contains various domains including arginine/serine (RS) rich‐domain, a G‐patch domain, and a double‐stranded RNA‐binding motif. RS domain is involved in protein–protein interactions and RNA processing.^3^, ^4^, ^5^, ^6^, ^7^, ^8^ Recently, Dingemans et al.^8^ have collected 52 cases classified as ZTTK syndrome with variants in *SON* and in the same year 15 unrelated cases were reported by Kushary et al.^9^

We report here a young child affected by ZTTK syndrome with *SON* mutations. The child along with the classical anomalies of the ZTTK syndrome showed Chiari Malformation type 1 (CM type 1) and hydrocephalus. The aim of this study is to review the classical clinical features of the ZTTK syndrome and, to include CM type 1 and hydrocephalus disorders among the other cerebral abnormalities reported in this syndrome. Diagnosis of variant‐mutations in ZTTK syndrome should be considered in patients with congenital anomalies, hydrocephalus, and Chiari malformations.

## 2 CASE REPORT

**FIGURE 1:**
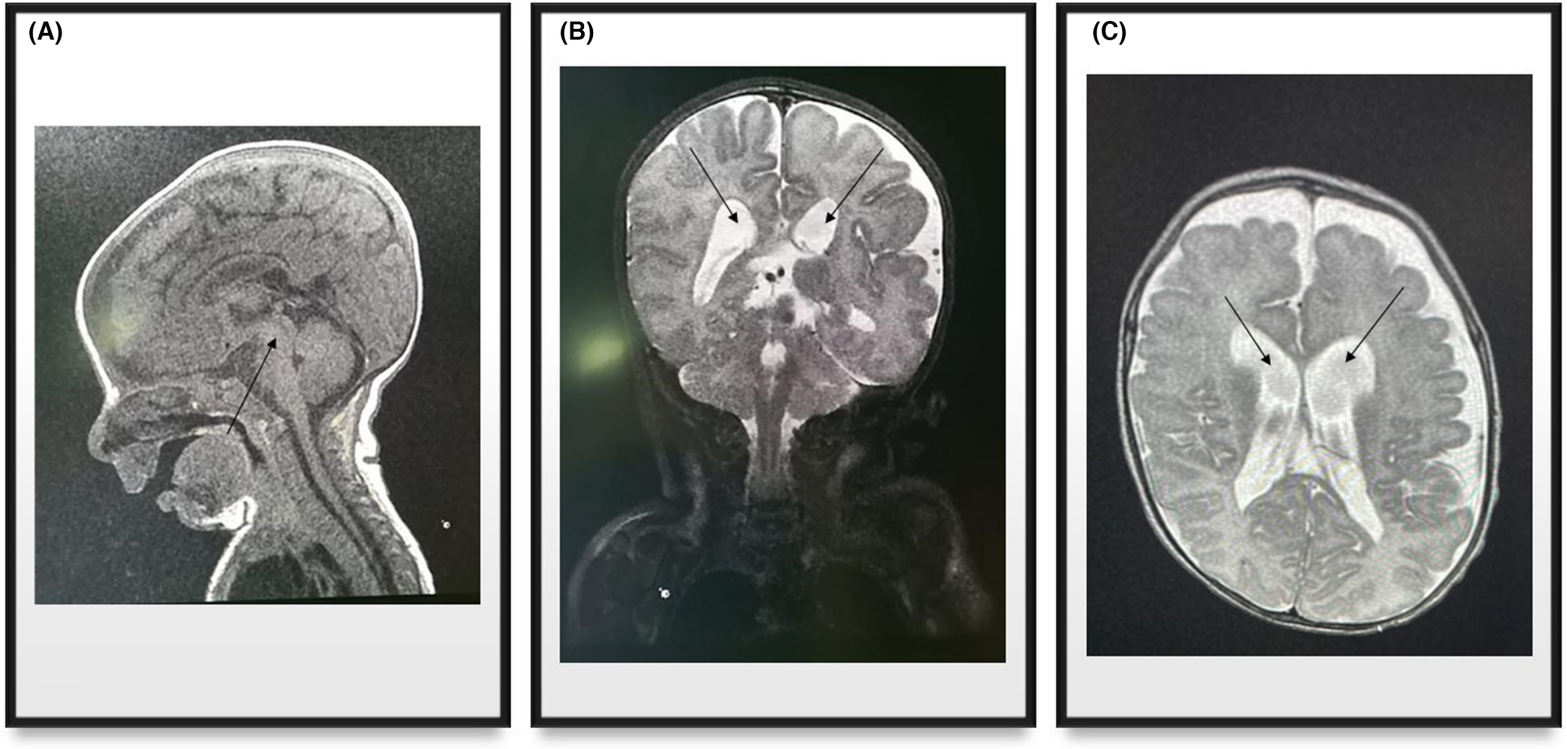
Brain MRI performed at the age of 1 month showing mild dilatation of lateral ventricles

This 2‐year‐old Caucasian boy is the first born of unrelated Italian parents. Family history is irrelevant and both parents are healthy with a good level of instruction, and no record of past or present relevant clinical disturbances. At the time of conception, the father was 33 years and the mother 27 years old. At the 8 months of gestation, the intrauterine ultrasound displayed reduction of the abdominal circumference and oligohydramnios. The child was born at 36 months of gestation by urgent cesarean section due to failure of induction, oligohydramnios, and intrauterine growth retardation (IUGR). The birth weight was 2.200, height 46 cm, occipitofrontal circumference (OFC) 31 cm. Apgar score was 8 at 1 min and 9 at 5 min. Some hours after birth he was admitted to a local Neonatal Intensive Care Unit (NICU) with signs of respiratory distress and expiratory moan. He was treated with CPAP ventilation for 72 h and then with oxygen at high‐flow therapy for 5 h. The presence of hypocalcemia and hyponatremia was corrected by supplementation. At the age of 1 month due to poor alimentation, hypotonia and craniofacial dysmorphism, brain, and spinal MRI was carried out with evidence of mild ventriculolateral dilation. (Figure 1). Electrocardiogram (ECG) and Echo‐ECG showed ostium secundum atrial septal defect with right cavity mildly dilated. He was hospitalized for 42 days and discharged with a diagnosis of preterm, small for gestational age (SGA), respiratory distress, hydrothorax, anemia, and mild cerebral ventriculolateral dilatation. In the following first months, the child showed a regular growth including OFC measurement, and delay in the stages of development with hypotonia and difficulty to maintain the sitting position at the age of 9 months. Physiotherapy treatment was started but irregularly conducted. At 10 months, the OFC was 47.5 cm (75th), and at 11 months 50 cm (<90th). At the age of 12 months during a routine pediatric examination for motor delay, the pediatrician noted anterior fontanelle very tense and bulging and the child was transferred to Emergency Care to Garibaldi Hospital, Catania. A prompt CT scan displayed the presence of triventricular hydrocephalus, paraventricular hypodensity, and trigonocephaly. As required by the parents, the child was rapidly transferred to the Neurosurgery Section, Gaslini Institute, Genoa for hydrocephus treatment. At admission, the brain MRI displayed abnormal dilation of the lateral ventricles and of III ventricle with associated notable atrophy of the deep white matter of the cerebral hemispheres and thinning of corpus callosum which appeared raised and arched due to ventricular enlargement. The residue white matter of centrum semiovale showed T2/Flair hypersignals compatible with gliosis. There was persistent cavum septum pellucidum with wide fenestration of the two septal lines. No evidence of structural malformations of cerebral cortex were found; there was flattening of the cerebral circumvolution due to ventricular dilation with normal representation of the spaces of the convexity. Sylvian acqueduct was pervious; IV ventricle was of little dimension with foramen of Luska and Magendie normally pervious. Cisterna magna was wide. Dynamic cerebrospinal fluid (CSF) study revealed flow reduction along the edge of foramen magnum. Brain MRI diagnosis was CM1 and chronic triventricular hydrocephalus. The child was submitted to endoscopic third ventriculocisternostomy with excellent result. Brain MRI performed after surgery showed signs related to the surgical treatment, and persistence of minimal caudal ectopia of cerebellar tonsils through the foramen magnum. Persistence of gliotic signs hyperintense in T2/Flair in residue white matter of centrum semiovale was reported. The Cisterna Magna was wide. CSF flow study showed the presence of regular signs of flow in the mesencephalic acqueduct and in the pre‐truncal area while in the post‐truncal area the flow liquoral was reduced. The child was discharged with diagnosis of hydrocephalus and CM1.

At the age of 14 months, he was admitted at the Child and Adolescent Neurology and Psychiatry of the Medical and Experimental Department of Catania University. His weight was 7.200 g. (>3th), height 73 cm (3th), OFC 50 cm (90th). General conditions were good. The child was alert, cheerful with good eye contact, and valid cry. He presented with minor, not specific craniofacial dysmorphisms consisting of high forehead, marked protrusion of the metopic suture (trigonocephaly) with underling flat angioma in the left forehead side. Mild sun setting phenomenon was present. The eyebrows were poorly present. The nose was short with anteverted naris. Micro‐retrognathia and precocious primary dentation were noted; ears were low set, anteverted, poorly structured and posteriorly rotated with short antitragus. Hammer forefinger, bilateral thumbs jerks, and bilateral partial syndactyly of the second and third toes were noticed. Testicles were retractile. Heart, thorax, abdomen, and internal organs were normal. Aside the flat angioma in the forehead, no others cutaneous anomalies were reported. Neurologic examination displayed marked hypotonia with difficulty to walk without support. Laboratory blood testing produced normal results, including plasma and urinary amino acids, organic acids, thyroid and celiac markers, and total cholesterol. The EEG at awake and during the sleep showed a slow background. ECG and Echo, and ophthalmologic examinations were normal including fundus oculi, abdominal and scrotal ultrasound, and otoacoustic emissions. At the last examination at 24 months old, he showed poor growth, developmental delay, and mild lower limbs hypertony. His weigh is 8.300 g (<3th), height 80 (3th), and OFC 50 cm (90th). Patellar tendon reflexes were present and brisk. Psychodiagnostics assessment (Griffiths Mental Development Scales III) gave the following results: basis of learning < 50; language and communication < 50; oculo‐manual coordination < 50; personal‐social–emotional < 50; gross motor < 50 (scales of development under the normal level).

## 3 GENETIC TESTING

The family was fully informed and provided signed informed consent. Genetic testing was performed. DNA sample of the proband and their parents was extracted from a peripheral blood sample using the standard phenol extraction protocol. Exome analysis of the proband and his parents were performed using SureSelect Human All Exon v6 (Agilent Technologies) and HiSeq 2500 platform (Illumina). Bioinformatics analysis of exome data from the propositus and his parents was performed using BEDTools, an open‐source software package comprised of multiple tools for comparing and exploring genomic datasets via fundamental “genome arithmetic” tasks.^10^ There was a heterozygous frameshift variant in *SON* gene of the proband NM_138927.4(SON): c.5767C > T (p.Arg1923Ter), which results in premature termination codon, thus predicted to cause the loss of the full‐length 2303 aminoacids protein due to truncation after first 1923 residues. The family study showed the absence of examined variants in probands' healthy parents indicating de novo events. Sanger sequencing was performed as independent confirmation. According to the guidelines of the American Society of Medical Genetics and Genomics (ASMG) in 2015, this novel variation meets the criteria to be identified as a likely pathogenic variant.

## 4 DISCUSSION

The main clinical features presented by this child mainly consisted of developmental and motor delay; minor, not specific craniofacial dysmorphisms including trigonocephaly with frontal flat angioma, curly hair, mild sun setting phenomenon, retro‐micrognathia, bilateral thumbs jerks, precocious primary dentation, and retractile testicles. At the age of 12 months, the signs of anterior fontanelle hypertension and the neuroradiological examinations led to the diagnosis of Chiari malformation type 1 (CM1) and hydrocephalus. The proband, aside CM1 and hydrocephalus showed at the brain MRI other cerebral anomalies including thin corpus callosum and signs of gliotic hyperintensity in the semioval center. Another case of a patient with a variant of the SON gene, CM1, and hydrocephalus as a similar phenotype was previously reported.^2^ An obstructive hydrocephalus indicated as “mild” has been also detected in another 13‐year‐old female patient.^11^ The clinical features presented by the proband led us to carry out a genetic analysis that disclosed a novel variant of the *SON* gene. The variation has not been found in the genetic analysis of the parents suggesting a de novo onset. The *SON* gene encodes a protein that contains protein binds RNA and promoter pre‐mRNA splicing, particularly of transcripts with poor splice sites. *SON* is involved in pluripotency and survival of embryonic stem cells and in the alternative splicing of other genes acting in epigenetic regulation and apoptosis causing neuronal migration defects and dendritic spine abnormalities.^3^, ^11^, ^12^ *SON* has been also reported to interfere in transcriptional regulation of hematopoietic process and tumorigenesis.^2^, ^3^, ^4^, ^9^ The clinical features of the ZTTK syndrome have been well documented. Tan et al.^13^ analyzing 28 cases of ZTTKs reported in the literature found the presence of ID in 28/28 (100%); facial dysmorphism in 28/28 (100%); brain malformations in 22/28 (79%); musculoskeletal abnormalities in 23/28 (82%); short stature in 18/28 (64%); hypotonia in 21/28 (75%); seizures in 13/28 (46%); eye and/or vision abnormality in 20/28 (71%); urogenital malformation in 9/28 (32%); and heart defect in 5/28 (18%). Yang et al.^11^ indicated the main clinical manifestations of the ZTTK syndrome in six points: (1) short stature; (2) DD and/or ID; (3) facial features showing facial asymmetry, frontal uplift, mid face depression, short nasolabial groove, low set ears, oblique cleft of eyes, sunken eyes, wide/low bridge of the nose, small mouth, thin upper lip, and high frontal arch and cleft palate; (4) combined various congenital malformations: (a) eyes, including cortical visual impairment, hyperopia, optic atrophy, and strabismus; (b) tooth dysplasia; (c) congenital heart disease; (d) gastrointestinal malformations and feeding difficulty; (e) urogenital system anomalies as single kidney, horseshoe and kidney dysplasia; (f) bone dysplasia of the spine and limbs, as small hands and feet, joint spasm, joint overextension, premature closure of cranial suture, scoliosis, kyphosis, and hemivertebra; (5) nervous system defects: hypotonia, delayed development, developmental regression, epilepsy, abnormal cerebral cortical gyration, ventriculomegaly, thin corpus callosum, arachnoid cyst, cerebellar dysplasia, and white matter abnormality; and (6) low level of immunoglobulin. In a recent report, Digemans et al.^8^ collected 52 cases of ZTTK syndrome including 18 previously unpublished and underline the variety of symptoms caused by *SON* haploinsufficiency which mostly involved neurological, musculoskeletal, developmental, visual, and congenital abnormalities. In the 41 subjects of this cohort in whom a brain imaging was performed, 37 (90%) of them presented with abnormalities of the brain mostly ventriculomegaly (24/41, 59%), cortical dysplasia (7/41, 17%), corpus callosum (18/41, 44%: mainly hypoplasia), cerebral white matter (6/41, 15%), and cerebellum (6/41, 15%).^8^ Also recent is the report of Kushary et al.^9^ who found in 15 cases the presence of DD/ID in all of cases (15 of 15, 100%) with most individuals (12 of 15, 80%) showing abnormal brain imaging mostly presenting with hypoplasia or agenesis of the corpus callosum (6 of 12, 50%) and ventricular enlargement (5 of 12, 41%). In addition, 8 of 12 (66%) had multiple findings at brain MRI. As upper mentioned, brain anomalies are consistent findings in subjects with *SON* mutations. Ventricular enlargement, thin corpus callosum, abnormal cerebral cortical gyration, arachnoid cyst, cerebellar dysplasia, and/or white matter abnormality were found in the report of Yang et al.^14^ in 85.9% of the subjects with this syndrome. Slezak et al.^15^ maintained that central nervous system defects were found in 86% of the subjects with ZTTK syndrome and widening of the ventricular system and abnormalities of the corpus callosum were the most frequent.

In Table 1, we summarized the clinical features of all patients presenting with *SON* mutations and classified as ZTTK syndrome.^2^, ^3^, ^7^, ^8^, ^9^, ^11^, ^13^, ^14^, ^15^, ^16^, ^17^, ^18^ Minor craniofacial dysmorphism and mild or severe cognitive impairment, brain abnormalities single or in association were almost constantly found.

**TABLE 1:** Clinical description of published reports of patients presenting with SON mutations and classified as ZTTK syndrome

Authors		^2^	^3^	^7^	^11^	^16^	^14^	^13^	^17^	^15^	^8^	^9^	^18^	Present case
Patient number		20	1	7	1	2	1	1	1	2	18	15	1	1
Gender		11 M/9 F	M	1 M/6 F	F	1 M/1 F	F	M	F	2 M	10 M/8 F	10 M/5 F	F	M
Dysmorphic features		20/20	+	7/7	+	−	+	+	+	2/2	18/18	13/14	+	+
Growth delay (height)		10/20	+	4/7	+	−	+	+	+	2/2	11/18	8/14	−	+
Neurological manifestations	DD/ID	20/20	+	7/7	+	−	+	+	+	2/2	15/16	15/15	+	+
	Hypotonia	15/20	+	5/7	+	−	−	+	−	2/2	7/18	13/15	+	+
	Seizures	11/20	−	3/7	−	−	−	+	+	1/2	8/18	9/15	+	−
Cerebral abnormalities	Microcephaly	5/15	−	3/7	−	−	−	−	−	0/2	6/16	8/15	−	−
	Macrocephaly	−	+	−	−	−	−	−	−	0/2	−	2/15	−	−
	Ventriculomegaly	14/19	−	3/6	−	−	−	+	−	1/1	6/13	7/15	+	−
	Hydrocephalus	−	−	−	+	−	−	−	−	−	−	−	−	+
	White matter	4/19	−	1/6	+	−	−	−	−	−	1/13	1/15	−	+
	Corpus callosum	10/19	−	2/6	+	−	−	+	−	−	3/13	6/15	−	+
	Cerebral structures	7/19	−	−	−	−	−	−	+	−	−	3/15	−	−
	Chiari type 1	1/19	−	−	−	−	−	−	−	−	−	−	−	+
	Cerebellar structures	4/19	−	−	−	−	−	−	−	−	2/13	−	−	−
Other anomalies	Skin‐hair‐nails	5/20	−	3/7	−	−	−	−	+	1/2	6/16	−	−	+
	Congenital heart disease	5/20	+	2/7	−	−	−	−	+	1/2	2/5	2/11	−	−
	Gastrointestinal	3/20	−	7/7	−	−	−	−	−	2/2	2/14	9/14	−	−
	Renal	2/20	−	2/7	−	2/2	−	+	−	1/2	8/14	−	−	−
	Ocular	15/20	−	5/6	−	−	−	−	+	2/2	10/18	7/12	+	−
	Hearing problems	3/20	−	−	−	−	−	−	−	0/2	4/18	−	−	−
	Urogenital	6/20	−	−	−	−	−	+	−	1/2	6/14	4/13	−	−

Abbreviations: “−”, (not reported/absent); “+”, (detected).

In conclusion, ZTTK syndrome is a severe multisystem developmental disorder presenting with facial dysmorphism, cognitive impairment, epilepsy, and congenital malformations affecting various organs including with high frequency the brain. CM1 and hydrocephalus may be recorded as clinical neurological manifestations of the *SON* mutations together with others cerebral and systemic organs anomalies. Data here presented showed that genetic analysis for *SON* mutations should be suggested in patients with craniofacial anomalies, DD/ID, and cerebral malformations including CM and hydrocephalus.

## AUTHOR CONTRIBUTIONS

PP and RR drafted the manuscript. XP, FS, AP, and MB contributed to the acquisition of data and literature review. RR, PP, and AP participated in constructive outline, discussions, and critical editing. All authors have read and agreed to the published version of the article.

## FUNDING INFORMATION

This research did not receive any specific grant from funding agencies in the public, commercial, or not‐for‐profit sectors.

## ETHICS STATEMENT

The consent has been obtained from parent's patient after full explanation of the purpose and nature of all procedures used.

## CONSENT

Written informed consent was obtained from the parent's patient to publish this report in accordance with the journal's patient consent policy.

## Data Availability

The data that support the findings of this study are available from the corresponding author upon reasonable request.
